# Effects of an empathy enhancement program using patient stories on attitudes and stigma toward mental illness among nursing students

**DOI:** 10.3389/fpsyt.2023.1304947

**Published:** 2024-01-03

**Authors:** Mi-Kyoung Cho, Mi Young Kim

**Affiliations:** ^1^Department of Nursing Science, Chungbuk National University, Cheongju, Republic of Korea; ^2^College of Nursing, Hanyang University, Seoul, Republic of Korea

**Keywords:** patient stories, empathy, communication, social distance, prejudice, mental illness, nursing student

## Abstract

**Objective:**

This study aims to explore the impact of an empathy intervention through patients’ stories and investigate its impact on attitudes and stigma toward mental illness among nursing students prone to hold prejudices against this condition.

**Methods:**

Using a quasi-experimental pretest-posttest design, this study focused on nursing students and examined the effects of an empathy enhancement program targeting individuals with mental illnesses on communication, social distance, and prejudice. Ninety third-year nursing students from S and C cities and H and C universities enrolled in psychiatric nursing courses participated in the study. The intervention lasted 4 weeks and used the patient’s story to facilitate a participatory approach to understanding the patient’s life and encouraging mutual growth and expansion of consciousness in the therapeutic relationship. Age was treated as a covariate and analyzed using a two-way repeated-measure analysis of covariance.

**Results:**

The Empathy Enhancement Program Using Patient Stories (EEP-PS) group and the clinical practicum group showed no significant differences in communication, social distance, and empathy scores between the two groups or across different time points. However, variations were observed when examining specific subdomains within each group and across time points. Informative communication (*F* = 10.34, *p* = 0.002) and affiliative communication (*F* = 21.60, *p* < 0.001), which are subcategories of communication, increased significantly in the posttest compared to the pretest. Among social distances, interpersonal-physical distance decreased significantly in the posttest compared to the pretest (*F* = 31.02, *p* < 0.001). Prejudice of incompetence (*F* = 6.52, *p* = 0.012) and prejudice of risk (*F* = 14.37, *p* < 0.001) were significantly lower in the posttest than in the pretest.

**Conclusion:**

Both the EEP-PS and clinical practicum groups experienced improvements in communication, social distance, and prejudice toward individuals with mental illness. This study suggests that direct patient interactions and the use of patient narratives as indirect methods are effective approaches for enhancing attitudes and reducing stigma toward mental illness among nursing students.

## 1 Introduction

Empathy is the ability to understand and accept another person’s position and perspective accurately (1). It is crucial for establishing therapeutic relationships with patients and providing care (2); therefore, fostering positive interactions between nurses and patients is critical. Moreover, interactions grounded in empathy, such as the one described, can significantly contribute to the mental health recovery of patients. Consequently, it is imperative for medical staff to cultivate empathetic relationships when providing treatment. However, the relationship between medical staff and patients is often more hierarchical in South Korea than in other countries. This clinical environment sometimes limits meaningful patient interactions, which can lead to decreased empathy toward patients owing to reduced sensitivity and awareness (3). True empathy requires a deep understanding of patients. One effective way to achieve this is to engage with the patient’s story, which expresses the physical, psychosocial, and economic challenges they face due to their illness (4). A patient’s story is often referred to as “illness narrative.” Through these narratives, healthcare professionals can obtain subjective data about the illnesses concerned and insights into the patient’s emotions and the significance of their disease experience (4, 5). This method helps healthcare providers establish genuine therapeutic relationships with patients (6), enhance their communication skills, broaden their understanding, and promote personalized patient care (7). Additionally, it provides healthcare professionals with an opportunity to introspect and reevaluate their professional identity (7).

In this context, healthcare professionals can delve into various narratives within a patient’s account of their illness experience, enabling a profound and comprehensive understanding of the phenomenon (8). Furthermore, illness narratives can be integrated seamlessly into educational settings (9, 10). Despite their potential, illness narratives are limited to certain areas. This highlights the urgent need to expand their use across various diseases and situations and assess the impact and value of embedding these narratives in educational programs.

Previous research has implemented illness narratives with medical or general college students, predominantly focusing on cancer and adopting the method of directly listening to patients (6, 11). However, it is difficult to invite patients into classroom settings due to various physical, temporal, and situational constraints. Consequently, when leveraging patients’ illness narratives, it is essential to explore alternatives to direct listening and assess their effectiveness.

Nursing students acquire hands-on experience in diverse clinical environments through clinical practicums. Through these placements, they refine their nursing roles and skills and cultivate their expertise to manage the nursing process, execute nursing interventions, and address on-site challenges specific to a patient’s nursing needs (12).

This study focuses on mental disorders not frequently encountered in daily life except in psychiatric practicum settings, such as schizophrenia. This disorder typically manifests during mid-to-late adolescence and persists throughout life. Schizophrenia places a substantial strain on patients, their families, and broader society. Diseases of this caliber often face severe discrimination and prejudice, negatively affecting patients and their families (13).

Unbiased knowledge of schizophrenia bolsters the resilience of patients’ families and facilitates adaptive coping mechanisms (14). Being informed about a disease promotes a positive outlook toward its patients (15). Therefore, nursing students must possess accurate knowledge of and constructive attitudes toward schizophrenia. This understanding could play a pivotal role in enhancing adaptive coping mechanisms and fostering positive patient interactions. Additionally, empathy has been underscored as a foundational element for establishing therapeutic relationships with patients (16). Hence, focusing on patients with mental disorders can be instrumental in demonstrating the role of empathy, particularly in scenarios involving communication barriers, social distancing, and prejudice.

Against this background, this study aims to develop the “Empathy Enhancement Program Using Patient Stories” (“EEP-PS”), drawing on interviews with psychiatric patients, framed within the context of essays and qualitative research about them. Our objective is to assess the effectiveness of this newly developed program. The target participants for the EEP-PS are nursing students who have completed theoretical coursework on mental disorders but have not yet participated in clinical practice. The outcomes of this group will be juxtaposed with those of the Clinical Practicum Group, which comprises nursing students who have undergone theoretical instruction and clinical practicum.

Students with clinical practicum experience may already have altered perceptions due to exposure to broad disease scenarios—irrespective of their direct interactions with psychiatric patients—regarding basic communication, social distance, and prejudice. Therefore, we have designated students without clinical practicum experience as the experimental group for the EEP-PS intervention (henceforth referred to as the “EEP-PS Group”). Their empathy levels were compared with those of the Clinical Practicum Group. The EEP-PS Group received the intervention, after which their empathy levels were compared with those of the Clinical Practicum Group.

## 2 Hypotheses

The hypotheses of this study are as follows:

1. The EEP-PS and clinical practicum groups will show No difference In communication scores.
1–1. The EEP-PS and Clinical Practicum groups will show no difference in informative communication scores.
1–2. The EEP-PS and Clinical Practicum groups will show no difference in affiliative communication scores.
1–3. The EEP-PS and Clinical Practicum groups will show no difference in authoritative communication scores.
2. The EEP-PS and clinical practicum groups will show No difference In social distancing scores.
2–1. he EEP-PS and Clinical Practicum groups will show no difference in interpersonal-physical distance scores.
2–2. The EEP-PS and Clinical Practicum groups will show no difference in interpersonal-social distance scores.
3. The EEP-PS and Clinical Practicum groups will show no difference in prejudice scores.
3–1. The EEP-PS and Clinical Practicum groups will show no difference in prejudice of irrecoverability scores.
3–2. The EEP-PS and Clinical Practicum groups will show no difference in prejudice of incompetence scores.
3–3. The EEP-PS and Clinical Practicum groups will show no difference in prejudice of risk scores.

## 3 Methods

### 3.1 Design

This quasi-experimental study investigated the effects of the EEP-PS on nursing students.

### 3.2 Participants

Participants were third-year nursing students from H University in S City and C University in C City. departments that offer accredited four-year programs, adhering to a standardized nursing curriculum with broadly analogous processes. Based on a previous study by Kim (6), the required sample size was determined using an effect size of 0.6, *α* = 0.05, power (1 − β) = 0.80, and a two-tailed independent *t*-test. This calculation yielded a minimum sample size of 45 participants per group, amounting to a total of 90 participants. The EEP-PS Group comprised students from H University who had completed their theoretical coursework on mental disorders but had not yet begun their clinical practicum. Conversely, the Clinical Practicum Group comprised students from C University who had already completed their clinical practicum following theoretical courses on mental disorders. Among those who voluntarily expressed interest in participating, 45 students in each group were chosen on a first-come, first-served basis.

### 3.3 Variables

#### 3.3.1 Communication style

Communication style can be classified into three distinct categories: informative, affiliative, and authoritative. The informative communication style refers to a style where nurses offer education after sharing information related to the current state of the disease, medical tests, ongoing medications, and nursing treatments. The affiliative communication style is characterized by a nurse’s friendly attitude, interest in the patient, active listening, and empathy. The authoritative style is characterized by nurses providing unilateral instructions to patients using medical terminology during the nursing process, typically without considering the patient’s feedback or feelings. Nursing students’ interpersonal communication styles were assessed using a tool tailored for nurses by Jeong (17) after adapting it to nursing students. This 18-item instrument comprises three domains: informative (six items), affiliative (six items), and authoritative (six items). Each item is rated on a 5-point Likert scale (1 = not at all, 5 = very much), with a higher total score indicating a greater inclination toward the associated communication style. The tool’s reliability (Cronbach’s α) was not specified. It was 0.86 in Park and Lee (18). In this study, Cronbach’s α was 0.89 for the informative, 0.79 for the affiliative, and 0.62 for the authoritative communication style.

#### 3.3.2 Social distance

Social distance refers to the varied levels of sympathetic understanding among individuals (19). It reflects the degree of prejudice or subjective feelings people have toward diverse social groups (20). Social distance toward schizophrenia was assessed using a tool adapted from the Korean version by Kim (21). This 12-item tool comprises two subscales (six items each for interpersonal-physical distance and interpersonal-social distance) derived from Westie (22) four subscales (residential distance, position distance, interpersonal-physical distance, and interpersonal-social distance) used to assess social distance toward persons with disabilities. Each item was rated on a 5-point Likert scale (1 = very much so, 5 = not at all), with items 8, 10, and 11 reverse-scored. A higher total score indicated greater social distancing toward patients with schizophrenia. Cronbach’s alpha was 0.80 in Kim’s study (21) and 0.90 in this study.

#### 3.3.3 Prejudice

Prejudice refers to cognitive and emotional responses based on preconceived notions that represent a set of beliefs about the attributes of a particular group (23). Prejudice against individuals with mental disabilities refers to their perception of them as incompetent, dangerous, or incurable. The scale created by Kim (24) and later refined by Kim and Seo (25) was used to quantify prejudice against mental disorders. In the original version, this prejudice scale comprised four factors (recoverability, incompetence, danger, and identifiability) with 24 items. In our study, only 21 items from the three factors were utilized, excluding the identifiability factor. Each item was rated on a 5-point Likert scale (1 = not at all, 5 = very much so), with a higher score indicating stronger prejudice toward people with mental disabilities. In Kim and Seo’s study (25), Cronbach’s alpha was 0.78 for recoverability, 0.67 for incompetence, and 0.79 for danger. In this study, the respective values were 0.77, 0.88, and 0.81, with an overall Cronbach’s alpha value calculated at 0.91.

### 3.4 Implementation of “empathy enhancement program using patient stories”

The EEP-PS delves into the stories of patients with schizophrenia. This program was conceived in a prior study by Kim (6), which used illness narratives in nursing education, integrated autobiographical essays from patients with schizophrenia, and conducted qualitative interviews. The researcher curated a selection of 10 books featuring autobiographical essays on schizophrenia and five qualitative research studies on mental disorders. A panel of four experts, comprising three nursing professors and one psychiatrist, validated their suitability as educational materials. The program was structured into eight groups of five to six participants. They read selected essays and qualitative studies, highlighted poignant narratives, jotted down emergent questions, and engaged in discussions. This exploration and discussion format was conducted over four 50-min weekly sessions.

The contents of each session are as follows:

1. Session 1: Introduction to the program, sharing personal experiences and thoughts on schizophrenia using data from essays and qualitative research.
2. Session 2: Exploration and discussion of material from essays and qualitative research, with participants summarizing and presenting content.
3. Session 3: In-depth analysis and discussion of data from essays and qualitative research, concluding with a review, summary, and presentation of findings.
4. Session 4: Self-reflection and organization, focusing on personal insights, learned lessons, impactful moments, and individual biases.

### 3.5 Implementation of the clinical practicum program for the clinical practicum group

Practicums were conducted based on clinical placements. Students were assigned ward practicums for a total practicum duration of 4 weeks. Clinical practicums typically commence with preliminary learning about diseases followed by direct face-to-face interactions and nursing care for patients. This clinical practicum is a clinical practicum generally conducted in a nursing department, and involves hands-on experience dealing with patients in various departments. Throughout the practicum, students engaged in direct communication and interactions with patients. Their nursing processes were documented in case studies, and in-depth reflections on each patient were facilitated during conferences.

### 3.6 Data collection

In April 2023, during the course orientation for the psychiatric nursing practicum, the researcher outlined the research objectives, methodologies, procedures for completing the online questionnaire, and details of research participation. From April 26 to 30, 2023, the information sheet and the online consent form were made available on the e-Campus Blackboard. Participants were recruited on a first-come, first-served basis, and only those who responded “I agree” to the online consent form were considered, capping the number at 45. Both participating universities undergo a four-year nursing education accreditation evaluation and adhere to a standardized curriculum, which makes their respective curricula similar. The information sheet, aimed at third-year nursing students, highlighted essential details such as the right to decline participation, neither benefits nor disadvantages of participation, the posttest to be conducted after the four-week program, and the participants’ freedom to withdraw at any point. The survey data were collected for 1 week, from May 1 to 7, 2023, with the corresponding URL shared on the e-Campus Blackboard, accompanied by orientation materials for online survey completion. The EEP-PS was administered to the EEP-PS group for 4 weeks (May 8 to June 2, 2023) using essays from patients with schizophrenia and qualitative research data, while the Clinical Practicum Group participated in the clinical practicum program. The posttest data collection took place between June 5 and 11, 2023, and the postsurvey URL was provided on the e-Campus Blackboard.

### 3.7 Ethical considerations

Data collection commenced after obtaining approval from the Institutional Review Board of the affiliated educational institution (no. CBNU-202304-HR-0069). The instructor elucidated the research objectives, implementation procedures, and data collection methods. Following the posting of the information sheet and online consent form on the e-Campus Blackboard, the pre- and post-survey URLs were distributed to the initial 90 respondents (45 in each group) who expressed their consent to participate. It was clearly stated that participation was voluntary, and each participant was granted the right to either halt participation or abstain from responding to the survey. The researcher’s contact information was provided to ensure that the participants could address any queries or opt to withdraw from the study with the assurance of exclusion from data analysis. Additionally, the participants were informed that discontinuation or refusal to complete the survey would not result in any future academic or related repercussions.

### 3.8 Data analysis

Data were analyzed using IBM SPSS Statistics (version 27.0; IBM, Armonk, NY, United States). Participants’ characteristics were described using mean, standard deviation, frequency, and percentage. For group homogeneity testing, we employed the independent *t*-test and χ^2^-test for the participants’ characteristics. If cell frequency was below 5, Fisher’s exact test was used. Communication style, social distance, and prejudice were examined using independent *t*-tests. The Shapiro–Wilk test was used to assess the normality of continuous variables. After the implementation of the empathy enhancement program, the outcome variables—communication style, social distance, and prejudice—were analyzed using a two-way repeated measures analysis of covariance (ANCOVA), with age (which showed non-homogeneity) treated as a covariate. Reliability was determined using Cronbach’s α, with the significance level set at *p* < 0.05 for all statistics.

## 4 Results

### 4.1 Characteristics of the participants

The participants’ general characteristics are as follows: the average age was 22.12 ± 2.74 years; women constituted 83.3% (*n* = 75) of the participants; 35.6% (*n* = 32) were identified as religious. The psychological distance toward mental disorders, gauged on a 10-point numeric rating scale, had an average score of 5.67 ± 1.88. Notably, 53.3% of participants (*n* = 48) scored above 6 (Table 1).

**Table 1:** Participants’ characteristics and homogeneity test between the two groups (*n* = 90).

Characteristics		Total (*n* = 90)	Clinical practicum group (*n* = 45)	Empathy enhancement program group (*n* = 45)	Χ^2^ or *t* (*p*)
		n (%)			
Age	<22	66 (73.3)	26 (57.8)	40 (88.9)	11.14 (0.002)
	≥22	24 (26.7)	19 (42.2)	5 (11.1)	
	M **±** SD	22.12 **±** 2.74	23.31 **±** 3.32	20.93 **±** 1.12	4.56 (<0.001)
Sex	Male	15 (16.7)	8(17.8)	7 (15.6)	0.08 (0.777)
	Female	75 (83.3)	37 (82.2)	38 (84.4)	
Religion	No	58 (64.4)	29 (64.4)	29 (64.4)	0.00 (0.587)
	Yes	32 (35.6)	16 (35.6)	16 (35.6)	
Psychological distance against mental disorders	<6	42 (46.7)	24 (53.3)	18 (40.0)	1.61 (0.205)
	≥6	48 (53.3)	21 (46.7)	27 (60.0)	
	M **±** SD	5.67 **±** 1.88	5.44 **±** 2.01	5.89 **±** 1.75	−1.12 (0.266)

M, mean; SD, standard deviation.

### 4.2 Homogeneity of characteristics and the variables between the two groups

Except for the age difference between the EEP-PS and Clinical Practicum groups, with members of the latter being older (*t* = 4.56, *p* < 0.001), no significant differences were observed in the participants’ characteristics between the two groups, confirming their homogeneity (Table 1). Regarding the dependent variables (three types of communication style, social distance, and prejudice), no significant differences were found between the Clinical Practicum and EEP-PS groups, further establishing group homogeneity (Table 2).

**Table 2:** Homogeneity test of the variables between the two groups (*n* = 90).

Variables	Total (*n* = 90)	Clinical practicum group (*n* = 45)	Empathy enhancement program group (*n* = 45)	*t* (*p*)
	M **±** SD			
Informative communication	24.72 **±** 3.47	24.09 **±** 2.96	25.36 **±** 3.85	−1.75 (0.084)
Affiliative communication	23.42 **±** 3.24	22.82 **±** 2.69	24.02 **±** 3.65	−1.78 (0.079)
Authoritative communication	18.44 **±** 2.33	18.27 **±** 2.35	18.62 **±** 2.32	−0.72 (0.472)
Social distance	36.16 **±** 4.66	35.20 **±** 4.27	37.11 **±** 4.89	−1.98 (0.051)
Interpersonal-physical distance	17.40 **±** 4.87	16.78 **±** 4.71	18.02 **±** 5.00	−1.22 (0.228)
Interpersonal-social distance	18.76 **±** 2.38	18.42 **±** 2.03	19.09 **±** 2.66	−1.34 (0.185)
Prejudice against mental disorders	50.33 **±** 10.86	49.71 **±** 11.03	50.96 **±** 10.77	−0.54 (0.590)
Prejudice of irrecoverability	16.01 **±** 3.73	16.47 **±** 3.99	15.56 **±** 3.43	1.16 (0.249)
Prejudice of incompetence	17.36 **±** 5.14	16.80 **±** 5.54	17.91 **±** 4.71	−1.02 (0.308)
Prejudice of risk	16.97 **±** 4.53	16.44 **±** 4.39	17.49 **±** 4.65	−1.10 (0.276)

M, mean; SD, standard deviation.

### 4.3 Hypothesis testing

**Figure 1:**
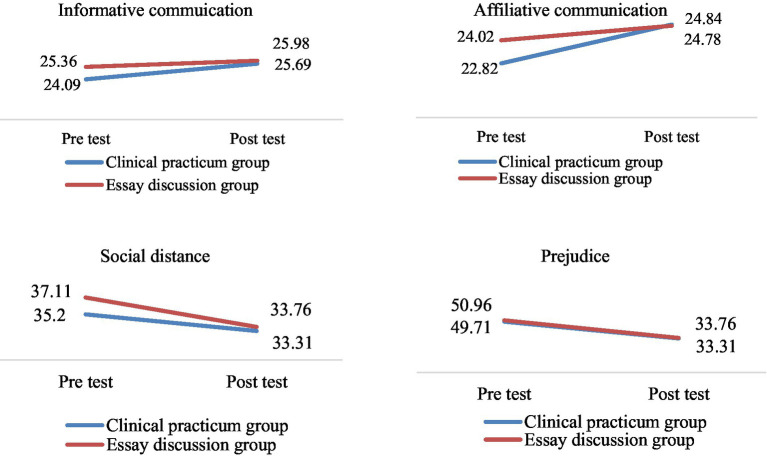
Differences in variables between the two groups (*n* = 90).

The variables measured after the program application were analyzed using a two-way repeated measures ANCOVA, with the mean age, which exhibited a difference between the EEP-PS and Clinical Practicum groups, set as a covariate (Table 3; Figure 1).

**Table 3:** Differences in variables between the two groups (*n* = 90).

Variables	Time	Clinical practicum group (*n* = 45)	Empathy enhancement program group (*n* = 45)		*F* (*p*)
		M **±** SD			
Informative communication	Pretest	24.09 **±** 2.96	25.36 **±** 3.85	Time	10.34 (0.002)
	Posttest	25.69 **±** 2.98	25.98 **±** 3.60	Group	0.69 (0.407)
				T*G	1.17 (0.283)
Affiliative communication	Pretest	22.82 **±** 2.69	24.02 **±** 3.65	Time	21.60 (<0.001)
	Posttest	24.84 **±** 3.18	24.78 **±** 3.36	Group	0.17 (0.680)
				T*G	1.89 (0.173)
Authoritative communication	Pretest	18.27 **±** 2.35	18.62 **±** 2.32	Time	0.24 (0.628)
	Posttest	18.27 **±** 2.35	18.62 **±** 2.32	Group	0.13 (0.720)
				T*G	-
Social distance	Pretest	35.20 **±** 4.27	37.11 **±** 4.89	Time	23.01 (<0.001)
	Posttest	33.31 **±** 4.64	33.76 **±** 4.83	Group	0.85 (0.360)
				T*G	1.95 (0.166)
Interpersonal-physical distance	Pretest	16.78 **±** 4.71	18.02 **±** 5.00	Time	31.02 (<0.001)
	Posttest	14.73 **±** 4.29	15.07 **±** 4.70	Group	1.03 (0.314)
				T*G	0.92 (0.341)
Interpersonal-social distance	Pretest	18.42 **±** 2.03	19.09 **±** 2.66	Time	0.24 (0.628)
	Posttest	18.58 **±** 1.91	18.69 **±** 2.36	Group	0.13 (0.716)
				T*G	1.77 (0.187)
Prejudice	Pretest	49.71 **±** 11.03	50.96 **±** 10.77	Time	12.14 (0.001)
	Posttest	33.31 **±** 4.64	33.76 **±** 4.83	Group	0.65 (0.424)
				T*G	1.06 (0.307)
Prejudice of irrecoverability	Pretest	16.47 **±** 3.99	15.56 **±** 3.43	Time	2.13 (0.148)
	Posttest	16.33 **±** 4.02	14.71 **±** 3.39	Group	1.07 (0.303)
				T*G	0.25 (0.621)
Prejudice of incompetence	Pretest	16.80 **±** 5.54	17.91 **±** 4.71	Time	6.52 (0.012)
	Posttest	16.07 **±** 5.33	16.02 **±** 4.76	Group	2.61 (0.110)
				T*G	1.67 (0.200)
Prejudice of risk	Pretest	16.44 **±** 4.39	17.49 **±** 4.65	Time	14.37 (<0.001)
	Posttest	15.47 **±** 3.60	15.80 **±** 4.22	Group	1.31 (0.256)
				T*G	0.07 (0.790)

F: Two-way repeated-measures ANCOVA; covariate: age. M, mean; SD, standard deviation; T, time; G, group; T × G, time × group.

*H1: The EEP-PS and Clinical Practicum groups will show no difference in communication scores.*

*H1-1: The EEP-PS and Clinical Practicum groups will show no difference in informative communication scores.*

No difference was found in the informative communication scores between the two groups and time by group interaction. However, a significant difference was observed over time (*F* = 10.34, *p* = 0.002). The Clinical Practicum Group showed a significant increase in the mean informative communication score post-program compared to pre-program (*F* = 8.72, *p* = 0.004), whereas the EEP-PS Group did not. Therefore, Hypothesis 1–1 is partially accepted.

*H1-2: The EEP-PS Group and Clinical Practicum groups will show no difference in affiliative communication scores.*

Regarding affiliative communication scores, we found no differences in time by group interaction between the EEP-PS and Clinical Practicum groups. However, a significant within-group difference was observed between the mean pre- and post-intervention scores (*F* = 21.60, *p* < 0.001). Both groups demonstrated a significant increase in affiliative communication post-intervention (EEP-PS: *F* = 4.35, *p* = 0.040; Clinical Practicum: *F* = 17.05, *p* < 0.001); therefore, Hypothesis 1–2 was partially accepted.

*H1-3: The EEP-PS and Clinical Practicum groups will show no difference in authoritative communication scores.*

Regarding authoritative communication scores, we found no differences between the EEP-PS and Clinical Practicum groups in terms of differences over time and time by group interaction. Furthermore, we observed no significant differences in the within-group mean scores between pre- and post-intervention tests. Therefore, Hypothesis 1–3 is accepted.

*H2: The EEP-PS and Clinical Practicum groups will show no difference in social distance scores.*

Regarding the social distance scores, while we found no difference in time by group interaction between the EEP-PS and Clinical Practicum groups, we observed a significant within-group difference between the pre- and post-intervention mean scores (*F* = 23.01, *p* < 0.001). Both groups showed a significant decrease in the mean social distance score (EEP-PS: *F* = 18.04, *p* < 0.001; Clinical Practicum: *F* = 4.70, *p* = 0.033). Therefore, Hypothesis 2 is partially accepted.

*H2-1: The EEP-PS and Clinical Practicum groups will show no difference in interpersonal-physical distance scores.*

Regarding the interpersonal-physical distance scores, although we found no significant intergroup difference in time by group interaction, we observed a significant within-group difference between the pre- and post-intervention mean scores (*F* = 31.02, *p* < 0.001). Both groups showed a significant decrease in the mean interpersonal-physical distance score (EEP-PS: *F* = 19.69, *p* < 0.001; Clinical Practicum: *F* = 9.07, *p* = 0.033). Therefore, Hypothesis 2–1 is partially accepted.

*H2-2: The EEP-PS and Clinical Practicum groups will show no difference in interpersonal-social distance scores.*

Regarding interpersonal-social distance scores, we found no differences between the EEP-PS and Clinical Practicum groups in terms of differences over time and time by group interaction. Furthermore, we observed no significant differences in the within-group mean scores between pre- and post-intervention tests. Therefore, Hypothesis 2–2 was accepted.

*H3: The EEP-PS and Clinical Practicum groups will show no difference in prejudice scores.*

Regarding the prejudice scores, although we found no differences between the EEP-PS and Clinical Practicum groups in terms of changes over time and time by group interaction, we observed a significant within-group difference between the pre- and post-intervention mean scores (*F* = 12.14, *p* = 0.001). Specifically, the EEP-PS group demonstrated a significant reduction post-intervention (*F* = 9.57, *p* = 0.003), whereas the Clinical Practicum group did not exhibit a notable decrease posttest. Therefore, Hypothesis 3 was partially accepted.

*H3-1: The EEP-PS and Clinical Practicum groups will show no difference in prejudice of irrecoverability scores.*

Regarding the prejudice of irrecoverability scores, we found no differences between the EEP-PS and Clinical Practicum groups in terms of differences over time and time by group interaction. Furthermore, we observed no significant differences in the within-group mean scores between pre- and post-intervention tests. Therefore, Hypothesis 3–1 was accepted.

*H3-2: The EEP-PS and Clinical Practicum groups will show no difference in prejudice of incompetence scores.*

Regarding the prejudice of incompetence scores, while we found no differences over time and time by group interaction between the EEP-PS and Clinical Practicum groups, we observed a significant within-group difference between the pre- and post-intervention mean scores (*F* = 6.52, *p* = 0.012). The EEP-PS Group demonstrated a significant post-intervention reduction (*F* = 7.12, *p* = 0.009), whereas the Clinical Practicum Group did not. Therefore, Hypothesis 3–2 is partially accepted.

*H3-3: The EEP-PS and Clinical Practicum groups will show no difference in prejudice of risk scores.*

Regarding the prejudice of risk scores, although we found no difference in the time by group interaction between the EEP-PS and Clinical Practicum groups, we observed a significant within-group difference between the pre- and post-intervention mean scores (*F* = 14.37, *p* < 0.001). Both groups exhibited a significant decrease in the post-intervention mean prejudice of the risk score (EEP-PS: *F* = 7.48, *p* = 0.008; Clinical Practicum: *F* = 5.46, *p* = 0.022). Therefore, Hypothesis 3–3 is partially accepted.

## 5 Discussion

This quasi-experimental study was conducted to examine the effects of the EEP-PS on nursing students. The null hypothesis testing of each variable is summarized as follows.

Regarding the three subcategories of communication pertaining to the first hypothesis, we observed the following:

(1) Informative communication: The EEP-PS Group did not show a significant increase post-intervention compared to pre-intervention, whereas a significant increase was observed in the Clinical Practicum Group. Informative communication strongly emphasizes the actual provision of information (including information about the patient’s condition), verifying that the patient understands the provided information and explaining the purpose of the medication and possible side effects (18). Conversely, EEP-PS focuses on the patient’s stories and the life of the subjects, which does not require concentration on the knowledge aspect. In this context, it would be difficult to enhance informative communication based on essays containing patients’ life stories or qualitative research alone. Contrarily, during a clinical practicum, participants meet the patient and study theory concurrently through case studies. They also review how to apply the learned theories in an educational context, which might explain the improved informative communication score in the Clinical Practicum Group.
(2) Affiliative communication: Both the EEP-PS and Clinical Practicum groups demonstrated a significant increase in affiliative communication post-intervention compared to pre-intervention. Affiliative communication, a subcategory of communication, is intrinsically linked to empathy. It encompasses genuinely listening to patients, sincerely understanding their pain, offering hope, and treating them with kindness (18). This indicates an enhancement in participants’ empathetic abilities, regardless of whether their exposure to patients was indirect through essays or direct interactions. This observation aligns with a previous study (8) highlighting that utilizing patients’ stories enables healthcare professionals to emotionally visualize patients’ circumstances. These vicarious experiences deepen our understanding of the patient’s experiential world. Moreover, the EEP-PS offers insights into diseases and engages participants in understanding a patient’s life by deciphering and delving into the nuances of their stories. Through this bond, both parties experience mutual evolution and expansion of consciousness (8), supporting the idea that patients’ stories are instrumental in enhancing the comprehension of their experiences.
(3) Authoritative communication: Neither the EEP-PS Group nor the Clinical Practicum Group demonstrated significant differences in scores from pre- to post-intervention. Authoritative communication refers to directing patients to follow instructions, cutting off patients’ stories, and dominating conversation (18). This finding suggests that interacting with patients—either directly or indirectly—does not foster or influence authoritative communication.

Regarding the second hypothesis concerning social distance, the EEP-PS and Clinical Practicum groups displayed a significant reduction in the mean social distance score post-intervention compared to pre-intervention. This indicates that either direct or indirect interactions with the patients can be instrumental in reducing social distance. Leveraging patient stories as an instructional method aids nursing students in cultivating a precise understanding and appropriate attitudes toward patients with mental disorders (26). This enhanced comprehension likely contributed to the observed reduction in social distance. Analyzing the subcategories, we noted differences between interpersonal-physical and interpersonal-social distance. The former group experienced a decrease in social distance post-intervention, whereas the latter remained relatively unchanged. Interpersonal-physical distance gauges perceptions of physical closeness through items such as the possibility of being genuine friends, traveling together, making home visits, or living as neighbors (27). Interpersonal-social distance evaluates aspects of social closeness using items such as the possibility of joining the same club, socializing frequently, or supporting others if they ran in an election (27). This latter category reflects individually perceived distance and poses the question of whether the wider society can accept them, necessitating broader social agreement and posing limits to narrowing this form of social distance.

Regarding the third hypothesis on prejudice, the EEP-PS Group showed a significant decrease post-intervention compared to pre-intervention, whereas the Clinical Practicum Group did not demonstrate a significant change. The analysis of the three subcategories revealed the following:

(1) Prejudice of irrecoverability: Both groups showed no significant difference from pre- to post-intervention, suggesting that neither group shifted to a more positive view of the recovery of mental disorders. The prejudice of irrecoverability refers to the belief that mental disorders are incurable, living a normal life post-recovery is difficult, lifelong treatment is required, relapse is likely, and treatment is time-consuming (28). It appears that patients’ stories do not change their perception of the chronic nature of mental disorders. In order to overcome the stigma associated with recovery, it is believed that it is necessary to include stories that include successful cases of community rehabilitation.

(2) Prejudice of incompetence: The EEP-PS Group showed a significant reduction of prejudice of incompetence post-intervention compared to pre-intervention. Prejudice of incompetence refers to the belief that individuals with mental disorders cannot distinguish right from wrong, manage themselves, make decisions independently, live without relying on others, or consistently adhere to social norms (27). Essays rich in detail and systematically presenting patient stories likely afforded readers an in-depth understanding of patients’ perspectives, subsequently dissolving many prevailing prejudices regarding their competence.

(3) Prejudice of risk: Both the EEP-PS and Clinical Practicum groups exhibited a decreased prejudice of risk post-intervention compared to pre-intervention. Prejudice of risk stems from the belief that individuals with mental disorders might be violent, could endanger children in their proximity, and are more prone to criminal activities than the general populace. Direct or indirect interactions with these patients appear to diminish such generalized fears and vague perceptions of potential danger (28). Notably, patients with schizophrenia, when under appropriate treatment, pose a minimal risk of criminal conduct; however, societal tendencies to stigmatize and discriminate based on inflated fears or biases can hinder their treatment processes (28). Thus, it is necessary to address and reduce unfounded fear and prejudice.

This study aimed to evoke emotional empathy using EEP-PS, a program designed to amplify empathy through patient stories. The results of this study indicate that integrating patients’ illness narratives into nursing education is one of the effective teaching-learning approaches to enhance students’ critical thinking and cognitive and ethical growth and foster individual autonomy in nursing practice. This synergy between cognitive and affective aspects means that it can be presented as one of the better teaching methodologies. Furthermore, this study emphasizes the importance of articulating and presenting patients’ stories in a written format. The study’s significance lies in its application of schizophrenia patients’ illness experience narratives to enhance empathy through education. In the future, extending education using patients’ disease experience narratives can go beyond mental disorders to encompass various medical conditions, particularly those associated with prejudice or rare diseases.

However, a limitation of this study is the absence of standardized processes for structuring and programming patients’ stories, cautioning against broad generalization of research results. Future developments, informed by this research, offer opportunities for creating and implementing diverse educational programs aimed at improving empathy.

## 6 Conclusion

This study empirically verified the efficacy of the EEP-PS and clinical practicum in enhancing empathetic communication and reducing social distance and prejudice toward patients with mental disorders. Both the EEP-PS and Clinical Practicum groups exhibited desired outcomes. Notably, despite using text as a medium to convey patients’ stories, the narratives effectively captured the essence of their lives, facilitating a deeper understanding. Hence, it is imperative to conduct further studies to validate the adaptability and impact of the EEP-PS across various disease contexts. In particular, when direct interactions with patients are impeded by physical or time constraints, leveraging written narratives can serve as a potent tool in nursing education to foster a richer comprehension of patients, which is contingent upon the program’s proven efficacy.

## Data availability statement

The raw data supporting the conclusions of this article will be made available by the authors, without undue reservation.

## Ethics statement

The studies involving humans were approved by the Chungbuk National University Institutional Review Board. The studies were conducted in accordance with the local legislation and institutional requirements. The participants provided their written informed consent to participate in this study.

## Author contributions

M-KC: Conceptualization, Data curation, Formal analysis, Software, Supervision, Validation, Visualization, Writing – original draft, Writing – review & editing. MK: Conceptualization, Data curation, Funding acquisition, Investigation, Methodology, Project administration, Resources, Validation, Visualization, Writing – original draft, Writing – review & editing.
